# Gas6/Axl Signaling Pathway in the Tumor Immune Microenvironment

**DOI:** 10.3390/cancers12071850

**Published:** 2020-07-09

**Authors:** Mai Tanaka, Dietmar W. Siemann

**Affiliations:** Department of Radiation Oncology, University of Florida, Gainesville, FL 32610, USA; siemadw@ufl.edu

**Keywords:** Gas6/Axl pathway, receptor tyrosine kinase, tumor immune microenvironment, immune evasion

## Abstract

Receptor tyrosine kinases have been shown to dysregulate a number of pathways associated with tumor development, progression, and metastasis. Axl is a receptor tyrosine kinase expressed in many cancer types and has been associated with therapy resistance and poor clinical prognosis and outcomes. In addition, Axl and its ligand growth arrest specific 6 (Gas6) protein are expressed by a number of host cells. The Gas6/Axl signaling pathway has been implicated in the promotion of tumor cell proliferation, survival, migration, invasion, angiogenesis, and immune evasion. As a result, Axl is an attractive, novel therapeutic target to impair multiple stages of tumor progression from both neoplastic and host cell axes. This review focuses on the role of the Gas6/Axl signaling pathway in promoting the immunosuppressive tumor microenvironment, as immune evasion is considered one of the hallmarks of cancer. The review discusses the structure and activation of the Gas6/Axl signaling pathway, GAS6 and AXL expression patterns in the tumor microenvironment, mechanisms of Axl-mediated tumor immune response, and the role of Gas6/Axl signaling in immune cell recruitment.

## 1. Introduction

Axl, also known as UFO, belongs in the Tyro3, MerTK, and Axl (TAM) subfamily of receptor tyrosine kinases. Axl and other TAM receptors can be activated via their ligands, growth arrest specific 6 protein (Gas6) and Protein S (Pros1), which are members of the family of vitamin K-dependent proteins. Axl is overexpressed in many cancer types and is associated with therapeutic resistance, poor clinical prognosis, and worse outcome [1,2,3,4]. Pre-clinical studies of Axl indicate that Axl mediates key components of the metastatic cascade, including but not limited to epithelial-to-mesenchymal transition, migration and invasion, proliferation, survival, stemness, and angiogenesis. In addition, soluble Axl (sAXL), an 80–85 kDa protein, is produced by the proteolytic cleavage of extracellular domains by A Disintegrin and Metalloproteinases 10 and 17 [5,6]. Increased serum levels of sAXL are associated with disease progression in a number of cancer types [4,7,8]. While the role of Gas6 and Axl in cancer has been broadly reviewed elsewhere [9,10], it is becoming increasingly clear that this signaling axis also impacts non-neoplastic cell populations which may be of particular interest when viewed in the context of the tumor microenvironment.

The importance of the tumor microenvironment in cancer development, progression, metastasis, and therapeutic resistance is now well recognized [11]. Furthermore, the tumor immune microenvironment (TIME) has gained significant attention over the last several decades, as neoplastic cells are able to promote the immunosuppressive microenvironment and evade immune surveillance. Indeed, the composition of the immune cells in the tumor microenvironment may predict clinical prognosis, therapeutic efficacy, and disease outcome [12]. An emerging factor in the modulation of the TIME is the Gas6/Axl signaling axis. This review focuses on the role of Gas6/Axl signaling in the tumor microenvironment, and its relation to potential mechanisms of immune evasion.

## 2. The Gas6/Axl Signaling Pathway

**Figure 1:**
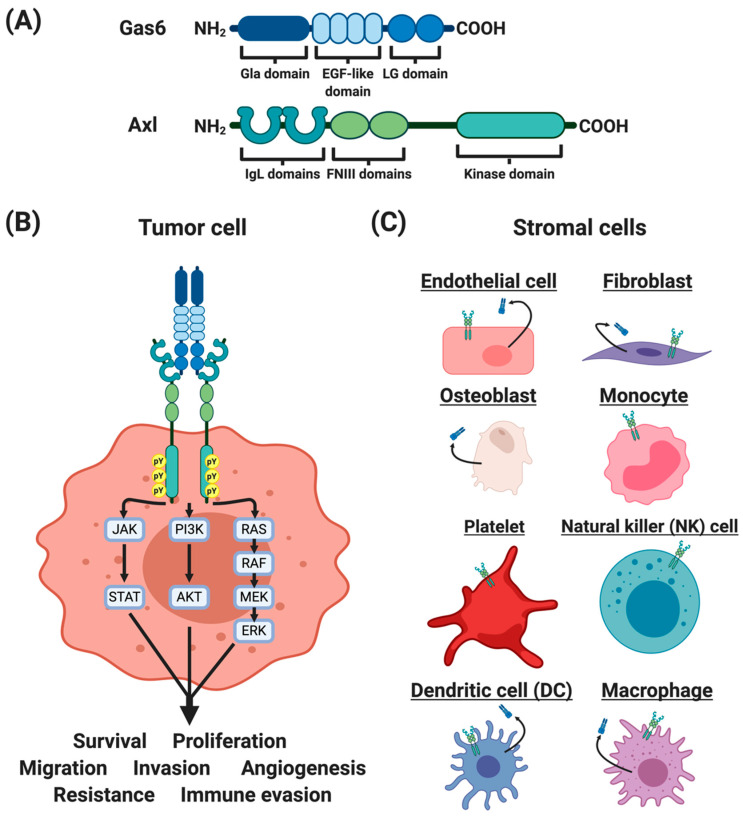
Structures and expression profiles of Gas6 and Axl. (**A**) The growth arrest specific 6 (Gas6) protein belongs in the family of vitamin K-dependent proteins. Gas6 consists of a gamma-carboxyglutamic acid (Gla) domain, four epidermal growth factor (EGF)-like domains, and two laminin G (LG)-like domains. Axl belongs in the Tyro3, Axl, MerTK (TAM) subfamily of the receptor tyrosine kinases. Axl consists of immunoglobulin-like (IgL) domains, two fibronectin domains, and a kinase domain. (**B**) Axl is expressed in a number of tumor types. The Gas6/Axl signaling promotes tumor cell survival, proliferation, migration, invasion, angiogenesis, therapeutic resistance, and immune evasion. (**C**) Gas6 and Axl are expressed by host stromal cells, including endothelial cells, fibroblasts, osteoblasts, monocytes, platelets, natural killer (NK) cells, dendritic cells (DCs), and macrophages.

AXL was first isolated from chronic myelogenous leukemia cells in 1988 [13] and characterized in 1991 [14,15]. Like all TAM receptors, Axl is composed of two immunoglobulin-like (IgL) domains, two fibronectin III (FNIII) domains, a transmembrane domain, and an intracellular kinase domain [15] (Figure 1A). The Axl protein contains 894 amino acids with a glycine-rich loop (Gly^543^- Gly^548^), a catalytic loop (His^670^-Asn^677^), and a DFG motif (Asp^690^-Phe^691^-Gly^692^). Although the molecular weight of the full-length Axl is 104 kDa, post-translational modifications of the extracellular domains give rise to two modified forms with molecular weights 120 and 140 kDa. Potential N-linked glycosylation sites include Asn^43^, Asn^157^, Asn^198^, Asn^339^, Asn^345^, and Asn^401^ [15].

Gas6 is one of the ligands for TAM receptors, with the highest affinity for Axl compared to Tyro3 or MerTK [16]. Gas6 was first identified by Schneider and colleagues in 1988 [17], and was characterized to be abundantly expressed in serum-starved 3T3 cells [17,18]. Gas6 contains 678 amino acids, with gamma-carboxyglutamic acid (Gla) domains (amino acids 49–90), four epidermal growth factor (EGF)-like domains (amino acids 118–278), and two laminin G-like (LG) domains (amino acids 279–678) [18,19] (Figure 1A). The N-terminus Gla domain mediates binding to cell membranes, particularly phosphatidylserine, in a calcium-dependent mechanism [20,21]. The crystal structures of Axl and Gas6 complex revealed that the C-terminus LG1 domain of Gas6 binds to the IgL-1 and IgL-2 domains of Axl [22,23]. Upon Gas6–Axl interaction, the complex dimerizes with another Gas6–Axl complex to form a 2:2 homodimerized complex with no direct Axl/Axl or Gas6/Gas6 contacts [22]. In addition, Axl has been reported to heterodimerize with other receptor tyrosine kinases, including EGFR [24,25], HER2 [26], HER3 [24], c-Met [27], and Tyro3 [28].

In the intracellular kinase domain of human Axl, tyrosine residues Tyr^698^, Tyr^702^, and Tyr^703^ are conserved among the TAM receptors. In addition, tyrosine residues (Tyr^779^, Tyr^821^, and Tyr^866^) interact with a number of signaling molecules, including phospholipase C (PLC), phosphatidyl inositol 3 kinase (PI3K), and Grb2 [29,30], and have been proposed as potential sites of autophosphorylation [15,29]. However, these conclusions must be viewed with caution as many of the earlier signaling studies used a chimeric EGFR/Axl receptor and both extracellular and intracellular domains impact the downstream signaling pathways [31,32]. Studies have demonstrated that genetic and pharmacologic inhibitions of Axl affect downstream signaling pathways including JAK-STAT, PI3K-AKT, and RAS-RAF-MEK-ERK [33,34].

## 3. GAS6 and AXL Expression in the Tumor Microenvironment

The tumor microenvironment consists of abnormal physiologic conditions, secreted factors, and host tumor-supporting cells, all of which play essential roles in cancer progression and metastasis [35]. While Axl expression on neoplastic cells is readily recognized, it is less well known that Axl is expressed by a variety of host cells found in the tumor microenvironment, including several immune cell types [36], fibroblasts [37], osteoclasts [38], and endothelial cells [39,40,41] (Figure 1B,C). Furthermore, the unique tumor microenvironmental conditions may modulate Axl and Gas6 expression in both neoplastic and host cells to promote aggressive and pro-tumorigenic phenotypes. For example, abnormal physiologic conditions such as low oxygen levels, or hypoxia, are a common occurrence in solid tumors and are known to be negative prognostic factors associated with disease progression and poor outcome [42]. Hypoxia upregulates hypoxia inducible factor-1 and -2 (HIF-1 and HIF-2), and modulates the expression of genes associated with angiogenesis, metabolism, cell survival, proliferation, motility, and invasiveness [43]. Several studies demonstrated that hypoxia upregulates and stabilizes Axl [4,44,45]. In addition to hypoxia, cytokines including transforming growth factor beta (TGF𝛽), granulocyte-macrophage colony stimulating factor (GM-CSF), and interferon-alpha (IFN⍺), have been shown to induce Axl expression [6,46]. Hence, the tumor microenvironment can modulate Axl expression in the various cell populations comprising tumors, to the point that Axl may be a critical mediator of the multimodal roles associated with tumor development, progression and metastasis

### 3.1. Axl Expression in Host Cells

Axl expression on endothelial cells is involved in mediating normal and tumor vasculature. For example, Axl inhibition decreases Tie2 and VEGFR-2 expression and impairs VEGF-A and lactate-induced activation of Akt [47,48]. In addition, Axl inhibition in tumor-bearing mice impairs tumor cell-induced angiogenesis and decreases immunohistochemical staining of the endothelial cell marker CD31 in the tumor [49,50,51,52]. Therefore, Axl inhibition impairs tumor cell-induced angiogenesis.

Axl expression on the cells of the human and murine immune system have been reviewed previously [53] (Figure 1C). Axl is primarily expressed by myeloid-lineage cells to phagocytose apoptotic cells and debris. Broadly, Axl is expressed on bone marrow derived cells (BMDCs) [54,55,56,57], dendritic cells (DCs) [6,36,58,59], macrophages [60,61], monocytes [56], natural killer (NK) cells [62], and platelets [63]. In addition, neoplastic cells may induce the expression of Axl and Gas6 in monocytic myeloid derived suppressor cells (M-MDSCs) and polymorphonuclear myeloid derived suppressor cells (PMN-MDSCs) [64]. Hence, the interactions between the neoplastic and host immune cells in the tumor microenvironment can potentiate the expression of Axl and Gas6 to promote a pro-tumorigenic microenvironment.

### 3.2. Gas6 Expression in Host Cells

In addition to neoplastic cells, Gas6 is expressed by luminal progenitor and basal cells around the ductal lining of the mammary tissue [65]. In the bone microenvironment, Gas6 is secreted by osteoblasts that are involved in forming bones [66,67,68]. Khoo and colleagues demonstrated that osteoblast-derived Gas6 induced Axl expression on neoplastic cells [68], suggesting that paracrine Gas6/Axl signaling promotes survival, inhibits apoptosis, and mediates homing of tumor cells to the bone.

In the tumor microenvironment, cancer-associated fibroblasts (CAFs) and CD45^+^-expressing tumor-infiltrating leukocytes (TILs) also express Gas6 [69,70,71,72]. Among the TILs, macrophages and dendritic cells express high levels of Gas6 [71,72], which can be further promoted in these cell types by IL-10, M-CSF, and IFN⍺ [6,72]. CD45^+^ cells from the bone marrow or the peripheral blood express significantly less Gas6 than TILs [72]. Although the mechanisms underlying Gas6 upregulation in TILs is not fully understood, in vitro studies demonstrate that tumor cells or tumor cell conditioned media induce Gas6 expression and secretion in macrophages [71,73]. Gomes and colleagues showed that stromal cell-derived Gas6 promotes tumor cell migration, invasion, survival, and proliferation [70,71]. Potential downstream effectors of the Gas6/Axl signaling through macrophage-derived Gas6 include pAkt and pStat3 [64,71]. In vivo, colorectal CT26 tumors grew slower in Gas6^−/−^ mice compared to wild-type (WT) mice [72]. In this model, transplanting bone marrow derived from Gas6^−/−^ mice into WT mice resulted in the slowing of tumor growth, suggesting that Gas6 from BMDCs supports tumor growth [72].

## 4. Mechanisms of Axl-Mediated Tumor Immune Response

**Figure 2:**
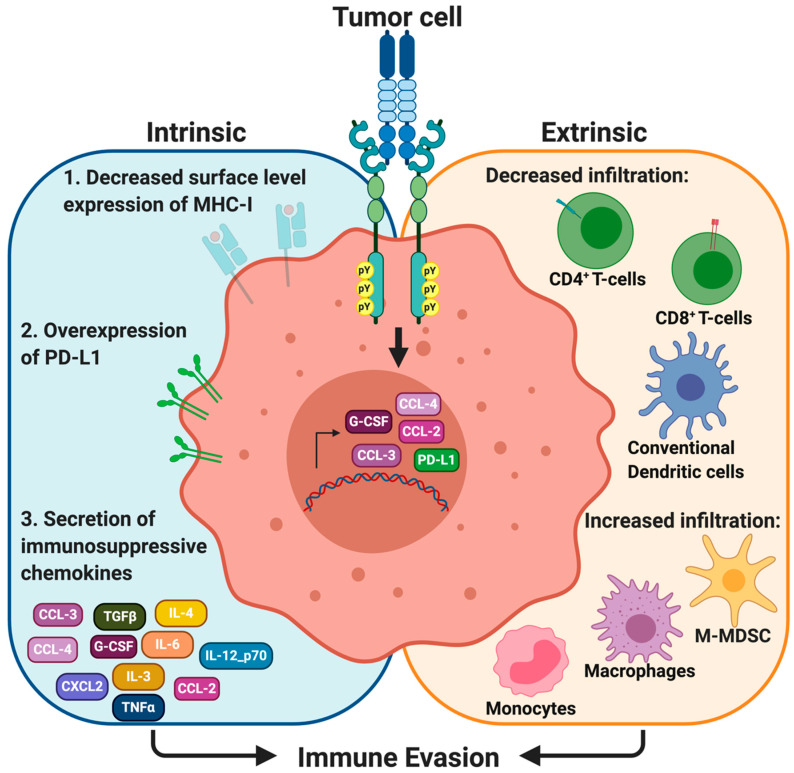
The Gas6/Axl signaling promotes the immunosuppressive tumor microenvironment. Axl signaling modulates surface level expression of major histocompatibility complex I (MHC-I) and programmed death ligand-1 (PD-L1) on neoplastic cells. The Gas6/Axl signaling also promotes secretion of immunosuppressive chemokines, including CCL3-5, G-CSF, IL-3, IL-4, IL-6, IL-12_p70, TGFβ, and TNFα. In the context of the tumor immune microenvironment, the Gas6/Axl signaling promotes infiltration of macrophages, monocytes, and myeloid-derived suppressor cells (MDSCs), but decreases infiltration of CD4^+^ and CD8^+^ T-cells, and conventional dendritic cells in the tumor.

The immune system plays an important role in cancer development, progression and metastasis [12], and it is becoming clear that both tumor intrinsic and extrinsic factors modulate the composition of the TIME [74,75]. Neoplastic cells alter the expression of cell surface molecules to avoid detection by surveilling immune cells. As such, a number of studies have revealed that Axl mediates key roles in promoting the immune suppressive tumor microenvironment (Figure 2).

### 4.1. Major Histocompatibility Complex Class I (MHC-I)

Major histocompatibility complex class I (MHC-I) molecules are present on the surface of all nucleated cells. When normal cells that are damaged, infected or considered ‘foreign’, MHC-I presents 8 to 11 amino acid-long epitopes derived from the MHC-I-expressing cell. These antigen-bound MHC-I complexes are recognized by circulating CD8^+^ T-cells and bind to the MHC-I complex by the CD8^+^/T-cell receptor complex. Upon binding, CD8^+^ T-cells are activated and secrete perforin and granzymes to lyse tumor cells. Hence, MHC-I presentation by neoplastic cells and other professional antigen presenting cells (APCs) is important for the eradication of tumor cells. An association between MHC-I and Axl was first observed by Rothlin and colleagues, who demonstrated that TAM knockout mice (Tyro3^−/−^Axl^−/−^MerTK^−/−^) had increased MHC-I-expressing myeloid cells [36]. More recently, studies by Guo and colleagues and Aguilera and colleagues independently showed that pharmacologic inhibition and genetic knockout of Axl, respectively, decreased surface level expression of MHC-I [76,77].

### 4.2. Programmed Cell Death Ligand 1 (PD-L1)

Neoplastic cells can escape immune surveillance and promote immune evasion through modulating the expression of cell surface receptors and ligands that differentiate between host and foreign cells. When neoplastic cells express immune checkpoint molecules, the host immune system recognizes these cells as ‘self’ instead of ‘foreign’, and prevents cell killing. Hence, immune checkpoint pathways have gained significant attention as potential therapeutic targets.

Programmed cell death ligand 1 (PD-L1) is one of the immune checkpoint molecules expressed in a number of tumor cell types. The interaction between PD-L1 with its receptor PD-1 on T-cells potentiates inhibitory signaling pathways to prevent T-cell activation [78]. This way, PD-L1-expressing neoplastic cells avoid immune-mediated cell death and continue to proliferate and survive in the TME (tumor microenvironment). Indeed, pharmacologic Axl inhibition using a selective Axl inhibitor, bemcentinib or BGB324, in lung adenocarcinoma cell lines (PC9 and H1975) significantly decreased PD-L1 and PD-L2, another ligand that binds to PD-1 [79]. Similarly, Axl knockdown in the human triple negative breast cancer cell line (MDA-MB-231) also decreased PD-L1 expression [80].

### 4.3. Altered Secretome

Axl signaling promotes an immune suppressive and pro-tumorigenic microenvironment through altered secretion of cytokines that modulate immune cell trafficking, migration, polarization, and adhesion [81,82] (Table 1). For example, a conditioned medium of an Axl knockout breast cancer cell line showed decreased secretion of granulocytic-colony stimulating factor (G-CSF) [77], which is known to promote accumulation of granulocytic-myeloid derived suppressor cells (G-MDSCs) in the TME [83]. Similarly, bemcentinib also decreased G-CSF in a genetically engineered mouse model of pancreatic cancer (Kras^LSL-G12D^; Cdkn2a^fl/fl^; Ptf1a^Cre/+^, KIC) [84]. Other studies have demonstrated that pharmacologic inhibition of Axl decreases IL-4 expression in the tumor [55,84], which promotes tumor progression and metastasis through mediating proliferation and survival of lymphocytes, and macrophage polarization towards the M2-like phenotype [85].

Chemokines are 8 to 10 kDa chemotactic cytokines that signal through seven transmembrane G protein-coupled receptors. Genetic and pharmacologic inhibitions of Axl impair secretion of chemokines involved in recruiting monocytes, macrophages, and M-MDSCs (CCL-2, CCL-3, CCL-4, and CCL-5) [76,77,84], and promote secretion of chemokines involved in recruiting Th1, CD8^+^ T-cells, and NK cells (CXCL9, CXCL10, and CXCL11) [76] (Figure 2).

Such altered cytokine secretion patterns in the tumor microenvironment may be associated with the suppressor of the cytokine signaling (SOCS) pathway. Physiologically, toll-like receptors (TLRs) are involved in innate immunity, particularly among dendritic cells and macrophages, as pattern recognition receptors. Activation of TLRs induces expression and secretion of proinflammatory cytokines. In dendritic cells, TLR activation also upregulates Axl [36]. The upregulated Axl is involved in the negative feedback regulation by forming a complex with a type I interferon receptor (IFNAR). Signaling through Axl–IFNAR induces the expression of the SOCS1 and SOCS3 that inhibit proinflammatory cytokine release and promote immunosuppression to maintain tissue homeostasis [36,86]. Hence, Axl mediates cytokine secretions by IFNAR, SOCS1 and SOCS3, at least in these in vitro immune cell cultures. However, whether these are downstream effectors of Axl signaling in the tumor cells remains unknown.

## 5. Involvement of the Gas6/Axl Signaling in Immune Cell Recruitment

Since the Gas6/Axl signaling pathway promotes an immunosuppressive TME, it is perhaps not surprising that the recruitment of specific immune cell types and the overall composition of the TIME also are altered (Figure 2). Immune cells can be broadly categorized as myeloid- or lymphoid- lineage cells that are involved in innate and adaptive immune responses, respectively. Both myeloid and lymphoid lineage cells collaborate to destroy foreign pathogens, including cancer cells. Still, tumor cells avoid detection and destruction by a number of immune cells, which is now considered one of the hallmarks of cancer [74].

Myeloid-lineage (CD11b^+^) cells consist of basophils, eosinophils, dendritic cells, mast cells, monocytes, macrophages, myeloid derived suppressor cells, natural killer cells, and neutrophils. Axl knockdown in a glioblastoma cell line decreased the percentage of tumor-infiltrating CD11b^+^ cells [87]. However, this was not observed in a pancreatic cancer model [84] (Table 2). Since CD11b^+^ cells represent a large group of cell types, this marker alone cannot predict the inflammatory status of the tumor. Of the CD11b^+^ cells, Guo and colleagues demonstrated that a selective Axl inhibitor, bemcentinib, decreased the number of infiltrating monocytes and macrophages in ID8 and 4T1 tumors [76] (Table 2). Similarly, in murine pancreatic cancer models, bemcentinib decreased tumor infiltrating macrophages [84,88].

CD11b^+^ cells found in the tumor immune microenvironment can be a double-edged sword in terms of cancer progression. While the infiltration of myeloid derived suppressor cells and tumor-associated macrophages (TAMs) promotes the immunosuppressive and pro-tumorigenic microenvironment, M1-like macrophages and dendritic cells can stimulate proinflammatory and anti-tumorigenic responses. Of the myeloid lineage cells, dendritic cells and macrophages are professional APCs that help to activate the adaptive immune response. Tumor infiltrating cDCs are thought to engulf dead- or dying- neoplastic cells or their cellular debris, process and present tumor-specific antigens through MHC-I or MHC-II, and mediate the cancer immunity cycle [89].

Lymphoid lineage cells consist of T-cells (thymus-derived), B-cells (bone marrow-derived), and natural killer T (NKT) cells. In particular, T-cells can be broadly categorized into CD8^+^ T-cells and CD4^+^ T-cells that recognize MHC-I and MHC-II on APCs, respectively. Hence, cells of the innate and adaptive immune system must work together to destroy neoplastic cells. Indeed, studies demonstrate that pharmacologic and genetic inhibitions of Axl increased the number of tumor-infiltrating cDCs [55,76,77,84] and the number of tumor-infiltrating CD4^+^ T-cells [55,76,77] (Table 2).

Among cDCs, CD103^+^ cDCs, but not CD11b^+^ cDCs, transport tumor-derived antigens to the tumor-draining lymph nodes and prime CD8^+^ T-cells for tumor cell lysis [90,91]. Guo and colleagues showed that bemcentinib-treated tumors increased CD103^+^ cDCs but not CD11b^+^ cDCs [76]. One study demonstrated that Axl inhibition increases the number of tumor-infiltrating CD8^+^ T-cells [92], while other studies demonstrated that Axl inhibition has no effect on the number of tumor-infiltrating CD8^+^ T-cells [55,77,87] (Table 2). Hence, further studies are warranted to understand the role of the Gas6/Axl signaling in the cancer immunity cycle.

## 6. Conclusions

The Gas6/Axl signaling pathway in neoplastic cells mediates multiple aspects of tumor progression and metastasis, including tumor cell proliferation, migration, invasion, survival, angiogenesis, therapeutic resistance, and immune evasion. Axl has been shown to be overexpressed in many cancer types, and is associated with poor clinical prognosis and outcome [9].

Yet, the tumor microenvironment consists of not only neoplastic cells, but also multiple normal host cell types that can support tumor growth, survival and metastasis. Crosstalk between neoplastic cells and immune cells can promote an immunosuppressive tumor microenvironment and immune evasion, which is considered one of the hallmarks of cancer [74]. These immune cells mediate tumor development, progression, and metastasis. Hence, the composition of the TIME may predict clinical prognosis, therapeutic efficacy, and disease outcome [12]. Indeed, the Gas6/Axl signaling pathway has been implicated in the promotion of the immunosuppressive tumor microenvironment and immune evasion through (1) altering surface level expression of MHC-I and PD-L1, (2) promoting secretion of immunosuppressive cytokines, and (3) escaping immune surveillance. Gas6 and Axl are also expressed by a number of host cells, including immune cells. Axl-expressing cDCs mediate cytokine secretions, and neoplastic cells upregulate Gas6 expression by the immune cells in the TME. Hence, both neoplastic and host cells found in the TME utilize the Gas6/Axl signaling pathway to promote aggressive tumor phenotypes.

Although this present review focuses on the role of the Gas6/Axl signaling pathway in the TIME, the other TAM family members Tyro3 and MerTK also are expressed by numerous neoplastic and host cells. For example, MerTK expression is elevated in immunosuppressive, M2-like macrophages compared to M1-like macrophages [93,94,95]. This macrophage polarization is, in part, potentiated by a Gas6-mediated mechanism [96]. Hence, the various TAM ligands and receptors may have overlapping roles that could further promote the immunosuppressive TIME. Our understanding of the Tyro3 and MerTK pathways will surely evolve as will the story of the Gas6/Axl signaling pathway in the TIME.

Given that Gas6 and Axl are expressed on both neoplastic and host cells, targeting this pathway presents a novel strategy to impair multiple stages of cancer development, progression, and metastasis. A number of Axl inhibitors have been developed and studied in preclinical and clinical settings for various cancer types [97]. As preclinical evidence pointing to the possible immunomodulatory roles of the Gas6/Axl pathway has evolved, clinical trials, such as the combination of the Axl inhibitor bemcentinib with pembrolizumab, have been initiated (ClinicalTrials.gov Identifier: NCT03654833, NCT03184558, NCT03184571). Indeed, understanding the Gas6/Axl signaling in the TIME undoubtedly will lead to the design of future therapeutic targeting strategies that may ultimately improve treatment outcomes.

## Figures and Tables

**Table 1:** Axl Signaling Modulates Secretion of Cytokines.

Changes in Cytokine Secretion upon Axl Inhibition		
Increased	Decreased	No Difference
**Colony stimulating factors**		
	CSF-1 [84], CSF-2 [77] ^b^, CSF-3 [77,84] ^b,c^	CSF-2 [84] ^c^
**Interleukin family**		
IL-12p40 [76] ^a^	IL-1a [77] ^b^, IL-3-5 [84] ^c^, IL-6 [77] ^b^, IL-9 [84] ^c^, IL-10 [76] ^a^, IL-12p40 [84] ^c^, IL-12p70 [84] ^c^, IL-13 [84] ^c^, IL-15 [84] ^c^, IL-17 [84] ^c^	IL-1a [84] ^c^, IL-2 [84] ^c^, IL-10 [84] ^c^, LIF [84] ^c^
**Chemokine family**		
CXCL9 [76] ^a^, CXCL10 [76] ^a^, CXCL11 [76] ^a^	CCL-2 [76,84] ^a,c^, CCL-3 [76,77,84] ^a,b,c^, CCL-4 [76,77,84] ^a,b,c^, CCL-5 [76,77,84] ^a,b,c^, CXCL1 [84] ^c^, CXCL2 [84] ^c^, CXCL5 [84] ^c^	CXCL12 [76] ^a^
**Other family of cytokines**		
INF𝛾 [76] ^a^	TGF𝛽 [76] ^a^, TNF𝛼 [77,84] ^b,c^	INF𝛾 [84] ^c^

Tumor types: ^a^ Ovarian and breast cancer models (ID8 and 4T1); ^b^ breast cancer cell line (Py8119); ^c^ pancreatic adenocarcinoma model, Kras^LSL-G12D^; Cdkn2a^fl/fl^; Ptf1a^Cre/+^ (KIC).

**Table 2:** Effects of Axl Inhibition on Tumor Infiltrating Immune Cells.

Tumor Infiltrating Immune Cell Markers	Identification Method	Effects of Axl Inhibition: Increased, Decreased, No Difference [Ref]
Leukocytes (CD45^+^)	FCM	Increased [76] ^a^ [77] ^b^
**T-cell subtypes**		
CD4^+^ T-cells (CD3^+^ CD4^+^ FoxP3^-^) (CD3^+^ CD4^+^)	FCM FCM	Increased [76] ^a^ No difference [77] ^b^
CD8^+^ T-cells (CD3^+^ CD8^+^)	FCM	Increased [76] ^a^ [77] ^b^
Tregs (CD3^+^ CD4^+^ FoxP3^+^)	FCM	No difference [76] ^a^ [77] ^b^
**Myeloid-lineage cells**		
Conventional dendritic cells (CD11b^+^ CD11c^+^ MHC-II^+^)	FCM	Increased [76] ^a^ [77] ^b^
Monocytes/Macrophages (CD11b^+^ F4/80^+^ Ly6G^-^)	FCM	Decreased [76] ^a^
TAM (CD11b^+^ Ly-6G^-^ Ly-6C^-^ F4/80^+^ CD11c^+^ MHC-II^+^)	FCM	Decreased [84] ^c^
Arg^+^ TAM (CD11b^+^ Ly-6G^-^ Ly-6C^-^ F4/80^+^ CD11c^+^ MHC-II^+^ Arg^+^)	FCM	Decreased [84] ^c^
Arg^+^ Macrophages (F4/80^+^ Arg^+^)	IF	Decreased [84] ^c^
Granulocytes (CD11b^+^ F4/80^-^ Ly-6G^+^)	FCM	Decreased [76] ^a^
M-MDSC (CD11b^+^ Ly-6G^-^ Ly-6C^+^)	FCM	Decreased [84] ^c^
PD-L1^+^ M-MDSC (CD11b^+^ Ly-6G^-^ Ly-6C^+^ PD-L1^+^)	FCM	Decreased [84] ^c^
PMN-MDSC (CD11b^+^ Ly-6G^+^ Ly-6C^+^)	FCM	No difference [84] ^c^
PD-L1 PMN-MDSC (CD11b^+^ Ly-6G^+^ Ly-6C^+^ PD-L1^+^)	FCM	No difference [84] ^c^

^a^ Guo and colleagues used murine ovarian and breast cancer models (ID8 and 4T1), and treated mice with 100 mg/kg bemcentinib (5 consecutive days/week). ^b^ Aguilera and colleagues used an Axl knockout murine breast cancer cell line (Py8119). The Axl knockout cell line was inoculated into wild-type syngeneic C57Bl/6 mice. ^c^ Ludwig and colleagues used murine pancreatic adenocarcinoma models (KPC-M09, Pan02, and KIC), and treated mice with 50 mg/kg bemcentinib twice daily. Abbreviations: Tregs—regulatory T-cells, TAM—tumor associated macrophages, M-MDSC—monocytic myeloid derived suppressor cell, PMN-MDSC—polymorphonuclear myeloid derived suppressor cell, FCM—Flow cytometry, IF—immunofluorescence.

## References

[1] Hutterer M., Knyazev P., Abate A., Reschke M., Maier H., Stefanova N., Knyazeva T., Barbieri V., Reindl M., Muigg A. (2008). Axl and growth arrest-specific gene 6 are frequently overexpressed in human gliomas and predict poor prognosis in patients with glioblastoma multiforme. Clin. Cancer Res..

[2] Shinh Y.-S., Lai C.-Y., Kao Y.-R., Shiah S.-G., Chu Y.-W., Lee H.-S., Wu C.-W. (2005). Expression of Axl in Lung Adenocarcinoma and Correlation with Tumor Progression. Neoplasia.

[3] Gjerdrum C., Tiron C., Høiby T., Stefansson I., Haugen H., Sandal T., Collett K., Li S., McCormack E., Gjertsen B.T. (2010). Axl is an essential epithelial-to-mesenchymal transition-induced regulator of breast cancer metastasis and patient survival. Proc. Natl. Acad. Sci. USA.

[4] Gustafsson A., Martuszewska D., Johansson M., Ekman C., Hafizi S., Ljungberg B., Dahlbäck B. (2009). Differential expression of Axl and Gas6 in renal cell carcinoma reflecting tumor advancement and survival. Clin. Cancer Res..

[5] Miller M.A., Oudin M.J., Sullivan R.J., Wang S.J., Meyer A.S., Im H., Frederick D.T., Tadros J., Griffith L.G., Lee H. (2016). Reduced Proteolytic Shedding of Receptor Tyrosine Kinases Is a Post-Translational Mechanism of Kinase Inhibitor Resistance. Cancer Discov..

[6] Scutera S., Fraone T., Musso T., Cappello P., Rossi S., Pierobon D., Orinska Z., Paus R., Bulfone-Paus S., Giovarelli M. (2009). Survival and migration of human dendritic cells are regulated by an IFN-alpha-inducible Axl/Gas6 pathway. J. Immunol..

[7] Dengler M., Staufer K., Huber H., Stauber R., Bantel H., Weiss K.H., Starlinger P., Pock H., Kloters-Plachky P., Gotthardt D.N. (2017). Soluble Axl is an accurate biomarker of cirrhosis and hepatocellular carcinoma development: Results from a large scale multicenter analysis. Oncotarget.

[8] Flem Karlsen K., McFadden E., Florenes V.A., Davidson B. (2019). Soluble AXL is ubiquitously present in malignant serous effusions. Gynecol. Oncol..

[9] Axelrod H., Pienta K.J. (2014). Axl as a mediator of cellular growth and survival. Oncotarget.

[10] Rankin E.B., Giaccia A.J. (2016). The Receptor Tyrosine Kinase AXL in Cancer Progression. Cancers (Basel).

[11] Dykes S.S., Hughes V.S., Wiggins J.M., Fasanya H.O., Tanaka M., Siemann D. (2018). Stromal cells in breast cancer as a potential therapeutic target. Oncotarget.

[12] Gajewski T.F., Schreiber H., Fu Y.X. (2013). Innate and adaptive immune cells in the tumor microenvironment. Nat. Immunol..

[13] Liu E., Hjelle B., Bishop J.M. (1988). Transforming genes in chronic myelogenous leukemia. Proc. Natl. Acad. Sci. USA.

[14] Janssen J.W., Schulz A.S., Steenvoorden A.C., Schmidberger M., Strehl S., Ambros P.F., Bartram C.R. (1991). A novel putative tyrosine kinase receptor with oncogenic potential. Oncogene.

[15] O’Bryan J.P., Frye R.A., Cogswell P.C., Neubauer A., Kitch B., Prokop C., Espinosa R., Le Beau M.M., Earp H.S., Liu E.T. (1991). Axl, a transforming gene isolated from primary human myeloid leukemia cells, encodes a novel receptor tyrosine kinase. Mol. Cell. Biol..

[16] Nagata K., Ohashi K., Nakano T., Arita H., Zong C., Hanafusa H., Mizuno K. (1996). Identification of the product of growth arrest-specific gene 6 as a common ligand for Axl, Sky, and Mer receptor tyrosine kinases. J. Biol. Chem..

[17] Schneider C., King R.M., Philipson L. (1988). Genes specifically expressed at growth arrest of mammalian cells. Cell.

[18] Manfioletti G., Brancolini C., Avanzi G., Schneider C. (1993). The protein encoded by a growth arrest-specific gene (gas6) is a new member of the vitamin K-dependent proteins related to protein S, a negative coregulator in the blood coagulation cascade. Mol. Cell. Biol..

[19] Bellido-Martin L., de Frutos P.G. (2008). Vitamin K-dependent actions of Gas6. Vitam. Horm..

[20] Huang M., Rigby A.C., Morelli X., Grant M.A., Huang G., Furie B., Seaton B., Furie B.C. (2003). Structural basis of membrane binding by Gla domains of vitamin K-dependent proteins. Nat. Struct. Biol..

[21] Mark M.R., Chen J., Hammonds R.G., Sadick M., Godowsk P.J. (1996). Characterization of Gas6, a member of the superfamily of G domain-containing proteins, as a ligand for Rse and Axl. J. Biol. Chem..

[22] Sasaki T., Knyazev P.G., Clout N.J., Cheburkin Y., Gohring W., Ullrich A., Timpl R., Hohenester E. (2006). Structural basis for Gas6-Axl signalling. EMBO J..

[23] Sasaki T., Knyazev P.G., Cheburkin Y., Gohring W., Tisi D., Ullrich A., Timpl R., Hohenester E. (2002). Crystal structure of a C-terminal fragment of growth arrest-specific protein Gas6. Receptor tyrosine kinase activation by laminin G-like domains. J. Biol. Chem..

[24] Taniguchi H., Yamada T., Wang R., Tanimura K., Adachi Y., Nishiyama A., Tanimoto A., Takeuchi S., Araujo L.H., Boroni M. (2019). AXL confers intrinsic resistance to osimertinib and advances the emergence of tolerant cells. Nat. Commun..

[25] Meyer A.S., Miller M.A., Gertler F.B., Lauffenburger D.A. (2013). The receptor AXL diversifies EGFR signaling and limits the response to EGFR-targeted inhibitors in triple-negative breast cancer cells. Sci. Signal..

[26] Goyette M.A., Duhamel S., Aubert L., Pelletier A., Savage P., Thibault M.P., Johnson R.M., Carmeliet P., Basik M., Gaboury L. (2018). The Receptor Tyrosine Kinase AXL Is Required at Multiple Steps of the Metastatic Cascade during HER2-Positive Breast Cancer Progression. Cell Rep..

[27] Salian-Mehta S., Xu M., Wierman M.E. (2013). AXL and MET crosstalk to promote gonadotropin releasing hormone (GnRH) neuronal cell migration and survival. Mol. Cell. Endocrinol..

[28] Brown J.E., Krodel M., Pazos M., Lai C., Prieto A.L. (2012). Cross-phosphorylation, signaling and proliferative functions of the Tyro3 and Axl receptors in Rat2 cells. PLoS ONE.

[29] Braunger J., Schleithoff L., Schulz A.S., Kessler H., Lammers R., Ullrich A., Bartram C.R., Janssen J.W. (1997). Intracellular signaling of the Ufo/Axl receptor tyrosine kinase is mediated mainly by a multi-substrate docking-site. Oncogene.

[30] Burchert A., Attar E.C., McCloskey P., Fridell Y.W., Liu E.T. (1998). Determinants for transformation induced by the Axl receptor tyrosine kinase. Oncogene.

[31] Fridell Y.W., Jin Y., Quilliam L.A., Burchert A., McCloskey P., Spizz G., Varnum B., Der C., Liu E.T. (1996). Differential activation of the Ras/extracellular-signal-regulated protein kinase pathway is responsible for the biological consequences induced by the Axl receptor tyrosine kinase. Mol. Cell. Biol..

[32] Linger R.M., Keating A.K., Earp H.S., Graham D.K. (2008). TAM receptor tyrosine kinases: Biologic functions, signaling, and potential therapeutic targeting in human cancer. Adv. Cancer Res..

[33] Gay C.M., Balaji K., Byers L.A. (2017). Giving AXL the axe: Targeting AXL in human malignancy. Nat. Publ. Group.

[34] Cruz V.H., Arner E.N., Du W., Bremauntz A.E., Brekken R.A. (2019). Axl-mediated activation of TBK1 drives epithelial plasticity in pancreatic cancer. JCI Insight.

[35] Quail D.F., Joyce J.A. (2013). Microenvironmental regulation of tumor progression and metastasis. Nat. Med..

[36] Rothlin C.V., Ghosh S., Zuniga E.I., Oldstone M.B., Lemke G. (2007). TAM receptors are pleiotropic inhibitors of the innate immune response. Cell.

[37] Espindola M.S., Habiel D.M., Narayanan R., Jones I., Coelho A.L., Murray L.A., Jiang D., Noble P.W., Hogaboam C.M. (2018). Targeting of TAM Receptors Ameliorates Fibrotic Mechanisms in Idiopathic Pulmonary Fibrosis. Am. J. Respir. Crit. Care Med..

[38] Nakamura Y.S., Hakeda Y., Takakura N., Kameda T., Hamaguchi I., Miyamoto T., Kakudo S., Nakano T., Kumegawa M., Suda T. (1998). Tyro 3 receptor tyrosine kinase and its ligand, Gas6, stimulate the function of osteoclasts. Stem Cells.

[39] Gallicchio M., Mitola S., Valdembri D., Fantozzi R., Varnum B., Avanzi G.C., Bussolino F. (2005). Inhibition of vascular endothelial growth factor receptor 2-mediated endothelial cell activation by Axl tyrosine kinase receptor. Blood.

[40] Fedeli C., Torriani G., Galan-Navarro C., Moraz M.L., Moreno H., Gerold G., Kunz S. (2018). Axl Can Serve as Entry Factor for Lassa Virus Depending on the Functional Glycosylation of Dystroglycan. J. Virol..

[41] Holland S.J., Powell M.J., Franci C., Chan E.W., Friera A.M., Atchison R.E., McLaughlin J., Swift S.E., Pali E.S., Yam G. (2005). Multiple roles for the receptor tyrosine kinase axl in tumor formation. Cancer Res..

[42] Vaupel P. (2008). Hypoxia and aggressive tumor phenotype: Implications for therapy and prognosis. Oncologist.

[43] Semenza G.L. (2003). Targeting HIF-1 for cancer therapy. Nat. Rev. Cancer.

[44] Rankin E.B., Fuh K.C., Castellini L., Viswanathan K., Finger E.C., Diep A.N., LaGory E.L., Kariolis M.S., Chan A., Lindgren D. (2014). Direct regulation of GAS6/AXL signaling by HIF promotes renal metastasis through SRC and MET. Proc. Natl. Acad. Sci. USA.

[45] Mishra A., Wang J., Shiozawa Y., McGee S., Kim J., Jung Y., Joseph J., Berry J.E., Havens A., Pienta K.J. (2012). Hypoxia Stabilizes GAS6/Axl Signaling in Metastatic Prostate Cancer. Mol. Cancer Res..

[46] Wilson C., Ye X., Pham T., Lin E., Chan S., McNamara E., Neve R.M., Belmont L., Koeppen H., Yauch R.L. (2014). AXL inhibition sensitizes mesenchymal cancer cells to antimitotic drugs. Cancer Res..

[47] Ruan G.X., Kazlauskas A. (2013). Lactate engages receptor tyrosine kinases Axl, Tie2, and vascular endothelial growth factor receptor 2 to activate phosphoinositide 3-kinase/Akt and promote angiogenesis. J. Biol. Chem..

[48] Ruan G.X., Kazlauskas A. (2012). Axl is essential for VEGF-A-dependent activation of PI3K/Akt. EMBO J..

[49] Lei X., Chen M., Nie Q., Hu J., Zhuo Z., Yiu A., Chen H., Xu N., Huang M., Ye K. (2016). In vitro and in vivo antiangiogenic activity of desacetylvinblastine monohydrazide through inhibition of VEGFR2 and Axl pathways. Am. J. Cancer Res..

[50] Kanlikilicer P., Ozpolat B., Aslan B., Bayraktar R., Gurbuz N., Rodriguez-Aguayo C., Bayraktar E., Denizli M., Gonzalez-Villasana V., Ivan C. (2017). Therapeutic Targeting of AXL Receptor Tyrosine Kinase Inhibits Tumor Growth and Intraperitoneal Metastasis in Ovarian Cancer Models. Mol. Nucleic Acids.

[51] Xiao Y., Zhao H., Tian L., Nolley R., Diep A.N., Ernst A., Fuh K.C., Miao Y.R., von Eyben R., Leppert J.T. (2019). S100A10 Is a Critical Mediator of GAS6/AXL-Induced Angiogenesis in Renal Cell Carcinoma. Cancer Res..

[52] Tanaka M., Siemann D.W. (2019). Axl signaling is an important mediator of tumor angiogenesis. Oncotarget.

[53] Rothlin C.V., Carrera-Silva E.A., Bosurgi L., Ghosh S. (2015). TAM receptor signaling in immune homeostasis. Annu. Rev. Immunol..

[54] Huey M.G., Minson K.A., Earp H.S., DeRyckere D., Graham D.K. (2016). Targeting the TAM Receptors in Leukemia. Cancers (Basel).

[55] Kasikara C., Davra V., Calianese D., Geng K., Spires T.E., Quigley M., Wichroski M., Sriram G., Suarez-Lopez L., Yaffe M.B. (2019). Pan-TAM Tyrosine Kinase Inhibitor BMS-777607 Enhances Anti-PD-1 mAb Efficacy in a Murine Model of Triple-Negative Breast Cancer. Cancer Res..

[56] Neubauer A., Fiebeler A., Graham D.K., O’Bryan J.P., Schmidt C.A., Barckow P., Serke S., Siegert W., Snodgrass H.R., Huhn D. (1994). Expression of axl, a transforming receptor tyrosine kinase, in normal and malignant hematopoiesis. Blood.

[57] Satomura K., Derubeis A.R., Fedarko N.S., Ibaraki-O’Connor K., Kuznetsov S.A., Rowe D.W., Young M.F., Gehron Robey P. (1998). Receptor tyrosine kinase expression in human bone marrow stromal cells. J. Cell. Physiol..

[58] Seitz H.M., Camenisch T.D., Lemke G., Earp H.S., Matsushima G.K. (2007). Macrophages and dendritic cells use different Axl/Mertk/Tyro3 receptors in clearance of apoptotic cells. J. Immunol..

[59] Subramanian M., Hayes C.D., Thome J.J., Thorp E., Matsushima G.K., Herz J., Farber D.L., Liu K., Lakshmana M., Tabas I. (2014). An AXL/LRP-1/RANBP9 complex mediates DC efferocytosis and antigen cross-presentation in vivo. J. Clin. Investig..

[60] Sharif M.N., Sosic D., Rothlin C.V., Kelly E., Lemke G., Olson E.N., Ivashkiv L.B. (2006). Twist mediates suppression of inflammation by type I IFNs and Axl. J. Exp. Med..

[61] Deng T., Zhang Y., Chen Q., Yan K., Han D. (2012). Toll-like receptor-mediated inhibition of Gas6 and ProS expression facilitates inflammatory cytokine production in mouse macrophages. Immunology.

[62] Paolino M., Choidas A., Wallner S., Pranjic B., Uribesalgo I., Loeser S., Jamieson A.M., Langdon W.Y., Ikeda F., Fededa J.P. (2014). The E3 ligase Cbl-b and TAM receptors regulate cancer metastasis via natural killer cells. Nature.

[63] Gould W.R., Baxi S.M., Schroeder R., Peng Y.W., Leadley R.J., Peterson J.T., Perrin L.A. (2005). Gas6 receptors Axl, Sky and Mer enhance platelet activation and regulate thrombotic responses. J. Thromb. Haemost..

[64] Holtzhausen A., Harris W., Ubil E., Hunter D.M., Zhao J., Zhang Y., Zhang D., Liu Q., Wang X., Graham D.K. (2019). TAM Family Receptor Kinase Inhibition Reverses MDSC-Mediated Suppression and Augments Anti-PD-1 Therapy in Melanoma. Cancer Immunol. Res..

[65] Mills K.L., Gomes A.M., Standlee C.R., Rojo M.D., Carmeliet P., Lin Z., Machado H.L. (2018). Gas6 is dispensable for pubertal mammary gland development. PLoS ONE.

[66] Shiozawa Y., Pedersen E.A., Patel L.R., Ziegler A.M., Havens A.M., Jung Y., Wang J., Zalucha S., Loberg R.D., Pienta K.J. (2010). GAS6/AXL axis regulates prostate cancer invasion, proliferation, and survival in the bone marrow niche. Neoplasia.

[67] Shiozawa Y., Pedersen E.A., Taichman R.S. (2010). GAS6/Mer axis regulates the homing and survival of the E2A/PBX1-positive B-cell precursor acute lymphoblastic leukemia in the bone marrow niche. Exp. Hematol..

[68] Khoo W.H., Ledergor G., Weiner A., Roden D.L., Terry R.L., McDonald M.M., Chai R.C., De Veirman K., Owen K.L., Opperman K.S. (2019). A niche-dependent myeloid transcriptome signature defines dormant myeloma cells. Blood.

[69] Kanzaki R., Naito H., Kise K., Takara K., Eino D., Minami M., Shintani Y., Funaki S., Kawamura T., Kimura T. (2017). Gas6 derived from cancer-associated fibroblasts promotes migration of Axl-expressing lung cancer cells during chemotherapy. Sci. Rep..

[70] Bae C.A., Ham I.H., Oh H.J., Lee D., Woo J., Son S.Y., Yoon J.H., Lorens J.B., Brekken R.A., Kim T.M. (2020). Inhibiting the GAS6/AXL axis suppresses tumor progression by blocking the interaction between cancer-associated fibroblasts and cancer cells in gastric carcinoma. Gastric Cancer.

[71] Gomes A.M., Carron E.C., Mills K.L., Dow A.M., Gray Z., Fecca C.R., Lakey M.A., Carmeliet P., Kittrell F., Medina D. (2019). Stromal Gas6 promotes the progression of premalignant mammary cells. Oncogene.

[72] Loges S., Schmidt T., Tjwa M., van Geyte K., Lievens D., Lutgens E., Vanhoutte D., Borgel D., Plaisance S., Hoylaerts M. (2010). Malignant cells fuel tumor growth by educating infiltrating leukocytes to produce the mitogen Gas6. Blood.

[73] Carron E.C., Homra S., Rosenberg J., Coffelt S.B., Kittrell F., Zhang Y., Creighton C.J., Fuqua S.A., Medina D., Machado H.L. (2017). Macrophages promote the progression of premalignant mammary lesions to invasive cancer. Oncotarget.

[74] Hanahan D., Weinberg R.A. (2011). Hallmarks of cancer: The next generation. Cell.

[75] Binnewies M., Roberts E.W., Kersten K., Chan V., Fearon D.F., Merad M., Coussens L.M., Gabrilovich D.I., Ostrand-Rosenberg S., Hedrick C.C. (2018). Understanding the tumor immune microenvironment (TIME) for effective therapy. Nat. Med..

[76] Guo Z., Li Y., Zhang D., Ma J. (2017). Axl inhibition induces the antitumor immune response which can be further potentiated by PD-1 blockade in the mouse cancer models. Oncotarget.

[77] Aguilera T.A., Rafat M., Castellini L., Shehade H., Kariolis M.S., Hui A.B., Stehr H., von Eyben R., Jiang D., Ellies L.G. (2016). Reprogramming the immunological microenvironment through radiation and targeting Axl. Nat. Commun..

[78] Keir M.E., Liang S.C., Guleria I., Latchman Y.E., Qipo A., Albacker L.A., Koulmanda M., Freeman G.J., Sayegh M.H., Sharpe A.H. (2006). Tissue expression of PD-L1 mediates peripheral T cell tolerance. J. Exp. Med..

[79] Tsukita Y., Fujino N., Miyauchi E., Saito R., Fujishima F., Itakura K., Kyogoku Y., Okutomo K., Yamada M., Okazaki T. (2019). Axl kinase drives immune checkpoint and chemokine signalling pathways in lung adenocarcinomas. Mol. Cancer.

[80] Kasikara C., Kumar S., Kimani S., Tsou W.I., Geng K., Davra V., Sriram G., Devoe C., Nguyen K.N., Antes A. (2017). Phosphatidylserine Sensing by TAM Receptors Regulates AKT-Dependent Chemoresistance and PD-L1 Expression. Mol. Cancer Res..

[81] Dranoff G. (2004). Cytokines in cancer pathogenesis and cancer therapy. Nat. Rev. Cancer.

[82] Chow M.T., Luster A.D. (2014). Chemokines in cancer. Cancer Immunol. Res..

[83] Waight J.D., Hu Q., Miller A., Liu S., Abrams S.I. (2011). Tumor-derived G-CSF facilitates neoplastic growth through a granulocytic myeloid-derived suppressor cell-dependent mechanism. PLoS ONE.

[84] Ludwig K.F., Du W., Sorrelle N.B., Wnuk-Lipinska K., Topalovski M., Toombs J.E., Cruz V.H., Yabuuchi S., Rajeshkumar N.V., Maitra A. (2018). Small-Molecule Inhibition of Axl Targets Tumor Immune Suppression and Enhances Chemotherapy in Pancreatic Cancer. Cancer Res..

[85] Li Z., Chen L., Qin Z. (2009). Paradoxical roles of IL-4 in tumor immunity. Cell Mol. Immunol..

[86] Yoshimura A., Naka T., Kubo M. (2007). SOCS proteins, cytokine signalling and immune regulation. Nat. Rev. Immunol..

[87] Sadahiro H., Kang K.D., Gibson J.T., Minata M., Yu H., Shi J., Chhipa R., Chen Z., Lu S., Simoni Y. (2018). Activation of the Receptor Tyrosine Kinase AXL Regulates the Immune Microenvironment in Glioblastoma. Cancer Res..

[88] D’Errico G., Alonso-Nocelo M., Vallespinos M., Hermann P.C., Alcala S., Garcia C.P., Martin-Hijano L., Valle S., Earl J., Cassiano C. (2019). Tumor-associated macrophage-secreted 14-3-3zeta signals via AXL to promote pancreatic cancer chemoresistance. Oncogene.

[89] Chen D.S., Mellman I. (2013). Oncology meets immunology: The cancer-immunity cycle. Immunity.

[90] Salmon H., Idoyaga J., Rahman A., Leboeuf M., Remark R., Jordan S., Casanova-Acebes M., Khudoynazarova M., Agudo J., Tung N. (2016). Expansion and Activation of CD103(+) Dendritic Cell Progenitors at the Tumor Site Enhances Tumor Responses to Therapeutic PD-L1 and BRAF Inhibition. Immunity.

[91] Roberts E.W., Broz M.L., Binnewies M., Headley M.B., Nelson A.E., Wolf D.M., Kaisho T., Bogunovic D., Bhardwaj N., Krummel M.F. (2016). Critical Role for CD103(+)/CD141(+) Dendritic Cells Bearing CCR7 for Tumor Antigen Trafficking and Priming of T Cell Immunity in Melanoma. Cancer Cell.

[92] Hua K.-T., Liu Y.-F., Hsu C.-L., Cheng T.-Y., Yang C.-Y., Chang J.-S., Lee W.-J., Hsiao M., Juan H.-F., Chien M.-H. (2017). 3′UTR polymorphisms of carbonic anhydrase IX determine the miR-34a targeting efficiency and prognosis of hepatocellular carcinoma. Sci. Rep..

[93] Shibata T., Habiel D.M., Coelho A.L., Kunkel S.L., Lukacs N.W., Hogaboam C.M. (2014). Axl receptor blockade ameliorates pulmonary pathology resulting from primary viral infection and viral exacerbation of asthma. J. Immunol..

[94] Zizzo G., Cohen P.L. (2018). Antibody Cross-Linking of CD14 Activates MerTK and Promotes Human Macrophage Clearance of Apoptotic Neutrophils: The Dual Role of CD14 at the Crossroads Between M1 and M2c Polarization. Inflammation.

[95] Myers K.V., Amend S.R., Pienta K.J. (2019). Targeting Tyro3, Axl and MerTK (TAM receptors): Implications for macrophages in the tumor microenvironment. Mol. Cancer.

[96] Kim S.Y., Lim E.J., Yoon Y.S., Ahn Y.H., Park E.M., Kim H.S., Kang J.L. (2016). Liver X receptor and STAT1 cooperate downstream of Gas6/Mer to induce anti-inflammatory arginase 2 expression in macrophages. Sci. Rep..

[97] Myers S.H., Brunton V.G., Unciti-Broceta A. (2016). AXL Inhibitors in Cancer: A Medicinal Chemistry Perspective. J. Med. Chem..

